# Diversity patterns of medicinal plants along elevational gradients across forest layers in Meihua Mountains, Fujian

**DOI:** 10.3389/fpls.2026.1856248

**Published:** 2026-05-29

**Authors:** Yu Hong, Yu Chen, Zhixin Chen, Lin Lin, Jinping Wu, Wei Xu, Liang Ma, Hai Ye

**Affiliations:** 1 Department of Pharmacy, Fujian Health College, Fuzhou, China; 2 Bureau of Meihua Mountain National Nature Reserve, Longyan, China

**Keywords:** diversity characteristics, elevational gradient, forest layers, medicinal plants, Meihua Mountains

## Abstract

**Introduction:**

Elucidating the diversity patterns and elevational distribution of medicinal plants is essential for biodiversity conservation and sustainable utilization in montane ecosystems. However, the variation in diversity across forest vertical layers and its responses to environmental gradients in mid-subtropical regions remain poorly understood.

**Methods:**

We surveyed medicinal plants in 93 plots across five elevational gradients (450–1800 m) in Meihua Mountain National Nature Reserve, China. Species composition and community structure in the tree, shrub, and herb layers were analyzed using importance values, *α*-diversity indices, and β-diversity metrics. Principal coordinate analysis (PCoA) and correlation analysis were performed to examine diversity patterns and identify their environmental drivers, respectively.

**Results:**

A total of 503 medicinal plant species (267 genera, 98 families) were recorded. Species richness followed the order shrub > herb > tree layer. Community composition showed clear elevational replacement. *α*-diversity declined overall with elevation, with significant unimodal patterns in shrub and herb layers but no clear trend in the tree layer. Species turnover was highest in the shrub layer and lowest in the tree layer, peaking at low to mid elevations. Elevation exerted stronger effects on *β*-diversity in shrub and herb layers than in the tree layer. Canopy closure was negatively correlated with shrub-layer *α*-diversity, whereas longitude and slope were positively associated with tree- and herb-layer diversity, respectively.

**Discussion:**

This study demonstrate that medicinal plant diversity in mid-subtropical mountains is jointly shaped by vertical stratification and environmental filtering. The higher sensitivity of shrub and herb layers highlights their key role in maintaining biodiversity, whereas tree-layer stability reflects greater resistance to environmental variation. Canopy structure regulates understory diversity, with additional layer-specific effects from topographic and spatial factors. These results provide new insights into diversity maintenance mechanisms and offer guidance for conservation and forest management of medicinal plant resources.

## 1 Introduction

Medicinal plants are an essential component of forest ecosystems and play a crucial role in maintaining species diversity and ecosystem stability (Sen and Samanta, 2014). They also constitute an important resource for traditional medicine and natural product development, with an estimated 350,000–500,000 plant species used globally, most of which are distributed in forest ecosystems (Salmerón-Manzano et al., 2020). The diversity and distribution patterns of medicinal plants not only reflect regional environmental conditions but also influence their conservation and sustainable utilization (Feng et al., 2023). However, wild medicinal plant resources have been continuously declining due to overharvesting, habitat fragmentation, and climate change (Shukla, 2023). This highlights an urgent need to clarify their spatial distribution patterns and underlying ecological drivers to support effective conservation and management.

Elevational gradients provide natural frameworks for examining biodiversity patterns (McCain and Grytnes, 2010), as they integrate multiple environmental factors, including temperature, precipitation, light, and soil conditions, over short geographic distances (Lomolino, 2001; He et al., 2023). Previous studies have reported diverse elevational patterns of plant diversity, such as monotonic decline, unimodal, or more complex relationships, depending on regional context and taxonomic groups (Dani et al., 2023; Zu et al., 2024). In particular, medicinal plant diversity has been shown to exhibit variable responses to elevation in different mountain systems. For example, unimodal patterns of species richness have been reported in the Himalayas (Rokaya et al., 2012), while other studies have identified weak or inconsistent elevational trends, especially across different forest layers (Zubair et al., 2021; Sudiana et al., 2025). These inconsistencies suggest that the effects of elevation on medicinal plant diversity are context-dependent and may vary with forest-layer structure and community composition. Despite growing interest in elevational biodiversity patterns, most studies have focused on overall plant communities, single vegetation layers (Spicer et al., 2021; Chen et al., 2025a) or specific species (Qian et al., 2022), with limited attention to vertical stratification of plant diversity. This tendency is also pronounced in studies of medicinal plants. Forest ecosystems are inherently structured into tree, shrub, and herb layers, each characterized by distinct life-history strategies, resource-use efficiencies, and sensitivities to environmental gradients (Gu et al., 2026). Ignoring this vertical heterogeneity may obscure important ecological processes governing diversity patterns (Stein et al., 2014). Moreover, while topographic factors such as slope and aspect are commonly considered, the role of forest structural attributes, particularly canopy closure, in shaping understory medicinal plant diversity remains insufficiently quantified (Balkrishna et al., 2024).

Meihua Mountain National Nature Reserve, located in the mid-subtropical region of southeastern China, represents a typical montane forest ecosystem with complex topography, high habitat heterogeneity, and well-developed vertical vegetation zonation. Although previous studies have documented general floristic composition and community diversity in this region (Kong and Li, 2012; Yu et al., 2026), systematic investigations of medicinal plant diversity and its elevational distribution across forest layers are still lacking. To address these gaps, this study conducted a comprehensive field survey of medicinal plants along an elevational gradient (450–1800 m) in Meihua mountain, incorporating tree, shrub, and herb layers. Specifically, we aimed to: (1) characterize the composition and vertical distribution of medicinal plant resources; (2) quantify *α*- and *β*-diversity patterns across elevational gradients and forest layers; and (3) identify the relative contributions of environmental and structural factors to diversity variation. By integrating vertical stratification with elevational analysis, this study seeks to advance understanding of diversity maintenance mechanisms in subtropical montane ecosystems and provide a scientific basis for the conservation and sustainable utilization of medicinal plant resources.

## 2 Materials and methods

### 2.1 Study area

**Figure 1:**
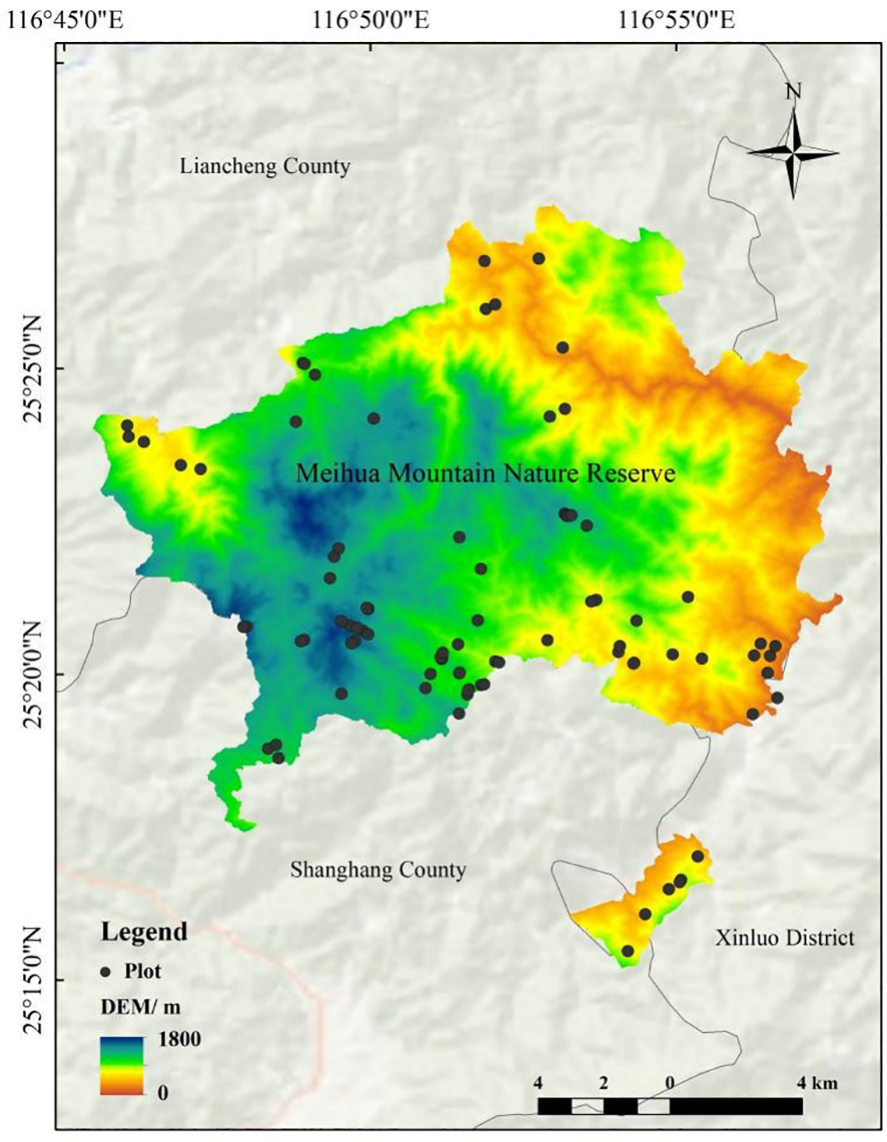
Distribution map of study area and sample plots.

The study was conducted in Meihua Mountain National Nature Reserve (25°15′14″–25°35′44″ N, 116°45′25″–116°57′33″ E), located in the Daimao Mountains between the southern Wuyi and Daiyun mountain ranges, Fujian Province, China (**Figure 1**). The reserve covers 22,168.5 hm² and is characterized by mountainous terrain, with higher elevations in the central and western regions and lower elevations toward the periphery. The mean elevation is approximately 900 m, with a maximum elevation of 1811 m and a relative elevation difference of 1436 m. The region has a typical subtropical maritime monsoon climate, with an annual mean temperature of 13–18 °C and annual precipitation ranging from 1700 to 2200 mm (Pan et al., 2023). Soils are primarily red soils, exhibiting clear vertical zonation, including mountain yellow-red soil, mountain yellow soil, and mountain meadow soil along the elevational gradient. Vegetation also shows distinct vertical zonation, comprising grassland, bamboo forest, evergreen broad-leaved forest, evergreen and deciduous broad-leaved mixed forest, mixed coniferous–broadleaved forest, and coniferous forest (Ling et al., 2025). Although Meihua Mountain is currently a protected area, some regions may have experienced low-intensity human disturbance in the past, such as selective harvesting of medicinal plants by local communities. However, no systematic or large-scale exploitation has been documented in recent decades, and the study sites are generally considered to represent relatively undisturbed forest conditions.

### 2.2 Plot setup and vegetation survey

Field surveys were conducted from October 2023 to October 2024. A total of 93 permanent plots (20 m × 20 m; total area 3.72 hm²) were established along an elevational gradient of 450–1800 m, covering eight vegetation types and 49 community formations, following the technical specifications of the Fourth National Census of Traditional Chinese Medicine Resources (**Table 1**). For each plot, geographic coordinates, elevation, slope, aspect, and habitat conditions were recorded. Vegetation surveys were conducted separately for tree, shrub, and herb layers using a nested sampling design based on plant growth forms rather than strictly defined taxonomic categories. Woody individuals with a single dominant stem (typically with a diameter at breast height (DBH) > 5 cm) and a height exceeding 3 m were classified as trees, whereas individuals lacking a distinct main stem, exhibiting multi-stemmed growth, or of shorter stature were classified as shrubs. For the tree layer, four 10 m × 10 m subplots were established within each plot. All individuals were recorded for species identity, height, DBH, and crown width. For the shrub layer, four 5 m × 5 m subplots were set at the corners of each plot to record species composition, plant height, basal diameter, individual number, and coverage. For the herb layer, four 1 m × 1 m subplots were used to record species identity, plant height, individual number, and coverage. Species identification and nomenclature followed the Angiosperm Phylogeny Group IV (APG IV) classification system (Group, 2016). Latin scientific names of medical plants were standardized and verified using the World Flora Online database (https://www.worldfloraonline.org/), with additional reference to the Flora of China (https://www.iplant.cn/foc). Medicinal plants were identified and classified based on standard references, including *Chinese Pharmacopoeia* 2025, *Medicinal Flora of China*, *Bencao Gangmu*, *Chinese Materia Medica*, as well as the Plant Photo Bank of China (PPBC) and relevant online databases.

**Table 1:** Basic information of sample plots at different elevational gradients.

Elevational gradient/m	Number of sample plot	Plant type (number)
450–750	20	Evergreen broad-leaved forest (3), Deciduous broad-leaved forest (1), Warm evergreen coniferous forest (4), Temperate coniferous forest (1), Grassland (7), Bamboo forest (4)
750–1000	20	Evergreen broad-leaved forest (10), Deciduous broad-leaved forest (1), Warm evergreen coniferous forest (1), Evergreen broad-leaved shrubland (1), Grassland (2), Bamboo forest (5)
1000–1250	18	Evergreen broad-leaved forest (3), Deciduous broad-leaved forest (2), Warm evergreen coniferous forest (7), Temperate coniferous forest (3), Evergreen broad-leaved shrubland (1), Grassland (1), Bamboo forest (1)
1250–1500	16	Evergreen broad-leaved forest (1), Deciduous broad-leaved forest (1), Temperate coniferous forest (10), Evergreen broad-leaved shrubland (1), Deciduous broad-leaved shrubland (1), Grassland (2)
1500–1800	19	Evergreen broad-leaved forest (3), Temperate coniferous forest (3), Evergreen broad-leaved shrubland (3), Bamboo forest (8), Grassland (2)

### 2.3 Importance value

The importance value (IV) was used to quantify the ecological dominance of species within each community (Xiao et al., 2024). For shrub and herb layers, IV was computed as:


$$IV=\frac{\mathrm{Relative}\;\mathrm{abundance}+\mathrm{Relative}\;\mathrm{frequency}+\mathrm{Relative}\;\mathrm{cover}}{3}$$

For the tree layer, relative dominance was used instead of relative coverage:


$$IV=\frac{\mathrm{Relative}\;\mathrm{abundance}+\mathrm{Relative}\;\mathrm{frequency}+\mathrm{Relative}\;\mathrm{dominance}}{3}$$

Species with IV greater than 5 in the tree layer and greater than 1 in the shrub and herb layers were defined as dominant species.

### 2.4 Diversity indices

#### 2.4.1 *α*-diversity

*α*-diversity was evaluated using the Margalef richness index (R), Shannon–Wiener diversity index (H), Simpson dominance index (D), and Pielou evenness index (J) (Thukral, 2017):


$$R=\frac{S-1}{ln\;N}$$

$$H=-\sum_{i=1}^{S}P_{i}\text{ln}P_{i}$$

$$D=1-\sum_{i=1}^{S}{\left(P_{i}\right)}^{2}$$

E=H/lnS

where *S* denotes the total number of species in the plot, *N* refers to the total number of individuals, and *P_i_* represents importance value of species *i*.

Data normality of *α*-diversity was first assessed using the Shapiro-Wilk test. When the assumption of normality was met, one-way analysis of variance (ANOVA) followed by least significant difference (LSD) tests was used to examine differences in *α*-diversity across elevational gradients (Wang et al., 2026). When normality was violated, the non-parametric Kruskal–Wallis test was applied instead (Nwobi and Akanno, 2021). All analyses were conducted separately for each forest layer (tree, shrub, and herb).

Additionally, the Spearman rank correlation analysis was used to examine the relationships between species diversity, environmental variables, and dominant species (Zar, 2005). Data normality was also assessed using the Shapiro–Wilk test prior to analysis. As most variables deviated from normality, and to ensure methodological consistency across all variables, Spearman rank correlation was employed instead of Pearson’s correlation.

#### 2.4.2 *β*-diversity

Species turnover along the elevational gradient was quantified using the Cody index (*β_C_*) (He et al., 2023):


$$\beta =\frac{a+b-2c}{c}$$

where *a* and *b* represent the total number of species in two plots, respectively, and *c* denotes the number of shared species between them.

Community compositional dissimilarity was further assessed using the Bray-Curtis index (*BC*) (Schroeder and Jenkins, 2018):


$$BC_{ij}=1-\frac{\sum_{k=1}^{S}min(BC_{ik},BC_{jk})}{\sum_{k=1}^{S}X_{ik}+\sum_{k=1}^{S}X_{jk}}$$

where *X_ik_* and *X_jk_* denote the relative abundance of species *k* in plot *i* and plot *j*, respectively. Principal coordinate analysis (PCoA) was used to visualize *β*-diversity patterns, and permutational multivariate analysis of variance (PERMANOVA) was applied to test for significant differences among elevational gradients (Huang et al., 2023).

### 2.5 Data analysis and visualization

All data calculations and statistical analyses, including importance value estimation (Section 2.3) and diversity indices (Section 2.4), were performed using Python 3.6. Data processing and analysis were conducted using the pandas, numpy and scipy libraries. Figures were generated using Python 3.6 (e.g., matplotlib and seaborn) and Microsoft Excel 2024.

## 3 Results

### 3.1 Composition and taxonomic structure of medicinal plants

A total of 503 medicinal plant species belonging to 267 genera and 98 families were recorded in the study area. Angiosperms dominated the flora, accounting for 449 species (235 genera, 81 families), followed by ferns (47 species, 26 genera, 11 families) and gymnosperms (7 species, 6 genera, 6 families). Distinct differences in taxonomic composition were observed among forest layers. The shrub layer exhibited the highest species richness (279 species, 143 genera, 72 families), followed by the herb layer (204 species, 148 genera, 67 families) and the tree layer (121 species, 71 genera, 43 families). The classification of forest layers is based on growth forms rather than mutually exclusive taxa. Therefore, some species (e.g., *Rhododendron championae*, *Castanopsis eyrei*, *Symplocos congesta*) may occur in multiple layers, resulting in non-additive species counts across layers. Evergreen species dominated both tree (76.0%) and shrub (60.2%) layers, whereas perennial herbs accounted for 75.0% of herb-layer species. Dominant families varied across layers. Lauraceae, Symplocaceae, and Fagaceae were most represented in the tree layer; Rosaceae, Myrsinaceae, and Lauraceae dominated the shrub layer; while Asteraceae, Poaceae, and Violaceae were prevalent in the herb layer. Across elevational gradients, taxonomic richness declined overall (**Table 2**). In the tree layer, the number of families decreased markedly at high elevation (1500–1800 m), with a reduction of 56.1% compared to mid-elevation levels. The shrub layer showed the highest richness at low elevation (450–750 m), followed by a continuous decline, with a 58.0% reduction at high elevation. In the herb layer, both genera and species richness decreased with elevation, particularly between 750–1000 m and 1000–1250 m.

**Table 2:** Statistics on medicinal plant resource types in different forest layers at different elevational gradients.

Forest layer	Type	Elevational gradient/m				
		450–750	750–1000	1000–1250	1250–1500	1500–1800
Tree	Family	14	13	14	14	11
	Genera/Species	55	41	44	42	18
Shrub	Family	21	19	20	18	12
	Genera/Species	138	113	128	101	58
Herb	Family	11	11	8	10	10
	Genera/Species	76	77	62	51	59

The genus-to-species ratio (G/S) of medicinal plants in each forest layer at different altitudinal gradients is approximately 1:1.

### 3.2 Elevational variation in dominant species and importance values

The importance values (VIs) of dominant species in all three layers shifted markedly with elevation (**Table 3**). In the tree layer, *Pinus hwangshanensis*, *Schima superba*, and *Cryptomeria japonica* var. *sinensis* showed increasing trend with rising elevation, whereas *Cunninghamia lanceolata* declined continuously. *Phyllostachys edulis* occurred across all elevations with consistently high IVs, indicating broad ecological adaptability. In the shrub layer, several species showed consistent directional changes with elevation. The IVs of *Eurya rubiginosa*, *Rhododendron simsii*, and *Pleioblastus amarus* increased with elevation, while *Camellia sinensis*, *Maesa japonica*, and *Rhododendron henryi* showed decreasing trends. Several species exhibited restricted elevational distributions, occurring only within specific zones. For example, *Rhododendron latoucheae* at 1250–1800 m and, and *Neolitsea aurata* and *Alniphyllum fortunei* at 450–1000 m. The herb layer shows the most complex variation in IV patterns. Some species (e.g., *Dicranopteris pedata*, *Woodwardia japonica*) exhibited unimodal trends, peaking at mid-elevation (1000–1250 m), whereas others (e.g., *Miscanthus floridulus*) declined monotonically with elevation. Several species (e.g., *Eremochloa ophiuroides*, *Oenanthe linearis*, and *Cibotium barometz*) were confined to single elevation bands, indicating strong habitat specificity.

**Table 3:** Importance values of dominant species in different forest layers at different elevational gradients.

Forest layer	Species	Elevational gradient/m				
		450–750	750–1000	1000–1250	1250–1500	1500–1800
Tree	*Phyllostachys edulis*	19.316	**27.794**	14.653	8.288	**29.781**
	*Cunninghamia lanceolata*	**21.746**	10.070	10.761	3.933	2.380
	*Pinus massoniana*	10.090	1.778	**18.209**	2.138	–
	*Castanopsis eyrei*	5.261	9.753	6.791	3.368	–
	*Pinus hwangshanensis*	–	0.568	–	**15.709**	27.733
	*Schima superba*	4.105	1.243	4.509	8.164	10.239
	*Cryptomeria japonica* var. *sinensis*	–	–	4.375	14.811	–
Shrub	*Rhododendron latoucheae*	–	0.558	–	**14.890**	**11.403**
	*Indocalamus tessellatus*	**11.868**	0.300	4.424	3.135	11.384
	*Schima superba*	3.576	1.951	5.274	4.364	4.439
	*Camellia sinensis*	3.848	**18.008**	9.437	0.718	1.848
	*Eurya rubiginosa* var. *attenuata*	–	0.907	1.185	3.476	11.327
	*Camellia oleifera*	1.086	2.230	9.437	0.269	1.848
	*Syzygium buxifolium*	0.503	2.137	**9.676**	–	–
	*Eurya nitida*	1.358	1.781	1.527	0.109	9.279
	*Rhododendron simsii*	2.414	2.143	7.633	**14.890**	**11.403**
	*Rhododendron championiae*	–	–	7.633	1.027	–
	*Pinus hwangshanensis*	–	0.097	–	2.789	6.694
	*Indocalamus latifolius*	1.777	–	4.424	3.135	–
	*Castanopsis eyrei*	0.743	3.103	1.730	2.510	–
	*Pleioblastus amarus*	–	–	2.982	–	4.717
	*Glochidion puberum*	–	–	0.403	–	7.460
	*Sarcandra glabra*	1.621	1.287	0.765	4.183	–
	*Dianthus chinensis*	8.669	–	–	–	–
	*Cunninghamia lanceolata*	2.452	1.624	1.759	1.159	–
	*Maesa japonica*	2.864	2.859	1.689	0.060	–
	*Adinandra millettii*	0.610	2.117	2.474	0.670	0.437
	*Machilus thunbergii*	0.321	1.256	2.128	1.514	0.221
	*Ardisia hanceana*	–	–	–	5.839	–
	*Neolitsea aurata*	0.109	4.369	–	1.961	–
	*Alniphyllum fortunei*	0.149	0.516	4.025	–	–
	*Rhododendron henryi*	2.414	2.143	0.607	0.294	0.140
Herb	*Dicranopteris pedata*	16.982	**14.647**	**34.857**	12.441	5.029
	*Miscanthus floridulus*	**26.693**	14.314	6.882	4.112	0.935
	*Miscanthus sinensis*	**26.693**	–	6.413	0.884	**24.908**
	*Diplopterygium glaucum*	1.745	5.319	6.594	**17.387**	0.966
	*Juncus effusus*	1.668	6.003	–	1.477	16.218
	*Woodwardia japonica*	2.936	4.594	7.730	4.167	0.623
	*Diplopterygium chinense*	1.425	13.502	5.053	–	1.234
	*Lophatherum gracile*	5.438	1.263	3.392	7.710	1.439
	*Eremochloa ophiuroides*	–	–	–	–	13.916
	*Reynoutria japonica*	–	0.837	–	11.311	–
	*Oplismenus undulatifolius*	0.659	–	3.551	2.399	1.452
	*Oenanthe linearis*	6.273	–	–	–	–
	*Utricularia bifida*	–	–	2.800	4.820	0.057
	*Selaginella doederleinii*	5.244	0.479	–	0.099	–
	*Kyllinga brevifolia*	–	–	–	7.908	–
	*Cibotium barometz*	4.217	–	–	–	–

“-” indicates that the importance value of the corresponding dominant species is unavailable, as the species was not observed at the specified altitude, making it impossible to calculate the importance value.

The tree layer lists species with an average importance value greater than 5, the shrub layer lists species with an average importance value greater than 1, and the herb layer lists species with an average importance value greater than 1.

The bold values represent the highest important value of medicinal plant species at each elevational gradient within each forest layer.

Based on the ranking of species importance values, five distinct tree–shrub–herb community assemblages were identified along the elevational gradient. From low to high elevation, these assemblages were: (1) *Cunninghamia lanceolata*-*Indocalamus tessellatus*-*Miscanthus floridulus* + *Miscanthus sinensis* (450–750 m); (2) *Phyllostachys edulis*-*Camellia sinensis*-*Dicranopteris pedata* (750–1000 m); (3) *Pinus massoniana*-*Syzygium buxifolium*-*Dicranopteris pedata* (1000–1250 m); (4) *Pinus hwangshanensis*-*Rhododendron latoucheae* + *Rhododendron simsii*-*Diplopterygium glaucum* (1250–1500 m); (5) *Phyllostachys edulis*-*Rhododendron latoucheae* + *Rhododendron simsii*-*Miscanthus sinensis* (1500–1800 m).

### 3.3 Patterns of *α*-diversity across elevational gradients

**Figure 2:**
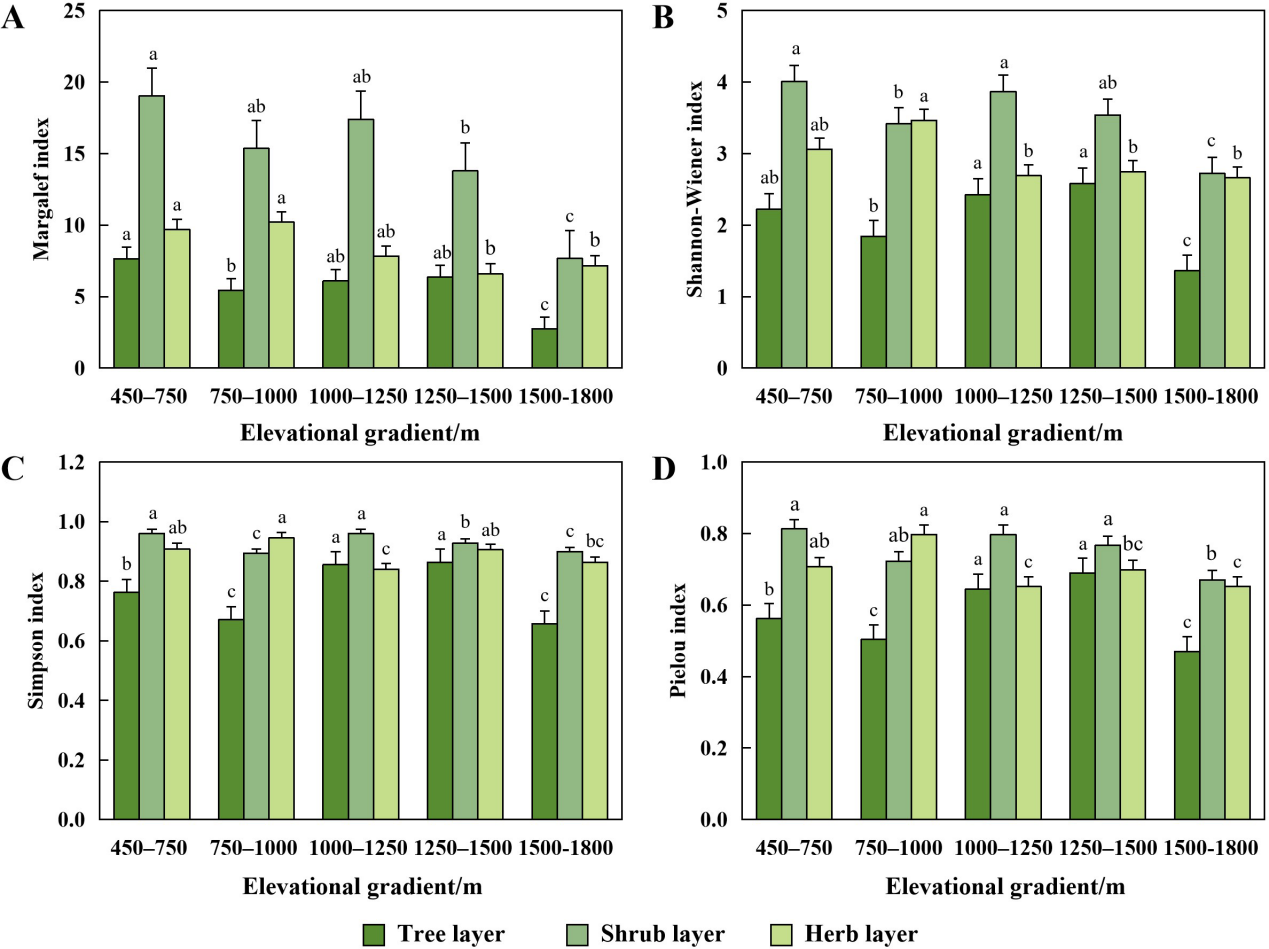
*α*-diversity indices of **(A)** Margalef, **(B)** Shannon-Wiener, **(C)** Simpson and **(D)** Pielou across forest layers at different elevational gradients. Different lowercase letters (a, b, ab, c, d) indicate significant differences among elevational gradients within each layer (P < 0.05), based on one-way ANOVA for normally distributed data or the Kruskal–Wallis test for non-normally distributed data.

*α*-diversity of medicinal plants exhibited clear variation along the elevational gradient, with distinct patterns among forest layers (**Figure 2**). Overall, *α*-diversity decreased with increasing elevation, although the magnitude and pattern of change differed among layers. Normality tests for all variables are presented in **Supplementary Table 1**. The Simpson and Pielou indices of both shrub and herb layers deviated from normality and all other indices conformed to normality. The shrub layer consistently showed the highest diversity, followed by the herb layer and the tree layer. In the shrub layer, all four diversity indices (Margalef, Shannon–Wiener, Simpson, and Pielou) displayed a clear declining trend with elevation. The highest values were recorded at low elevation (450–750 m), with Margalef index ranging from 7.66 to 19.02 and Shannon-Wiener index from 2.72 to 4.01. Diversity decreased progressively toward higher elevations. In the herb layer, *α*-diversity showed a unimodal tendency, with peak values at 750–1000 m. At this elevation, the Margalef, Shannon–Wiener, Simpson, and Pielou indices reached 10.20, 3.46, 0.94, and 0.80, respectively. Above this range, diversity declined with increasing elevation. In the tree layer, *α*-diversity was comparatively low and exhibited no consistent monotonic trend. The Margalef index peaked at low elevation (450–750 m; 7.65), whereas the Shannon–Wiener, Simpson, and Pielou indices reached maximum values at mid-to-high elevation (1250–1500 m; 2.58, 0.86, and 0.69, respectively), followed by a decline at the highest elevation.

**Figure 3:**
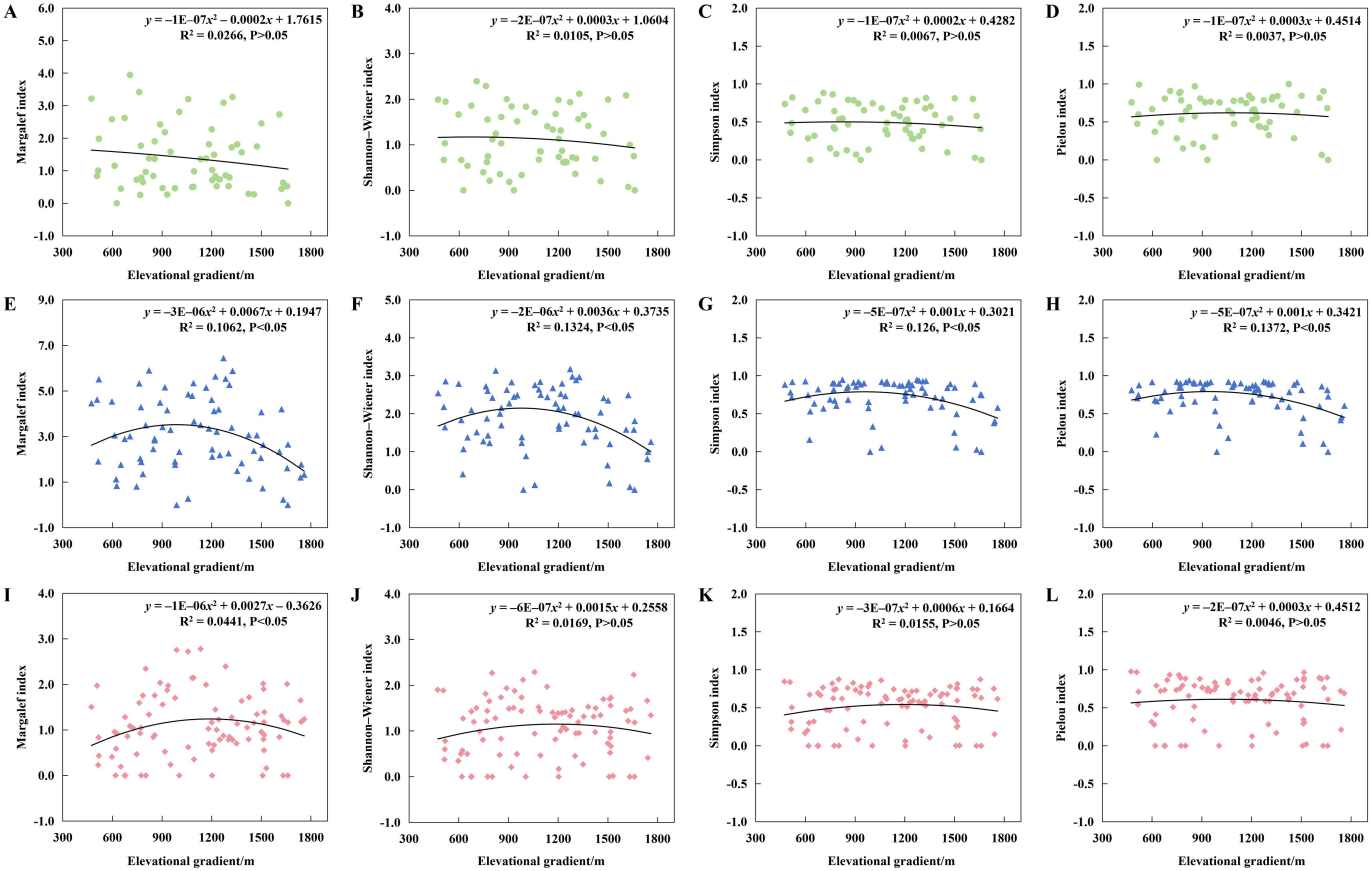
Fitting equations between *α*-diversity indices and altitude for medicinal plants in different forest layers. **(A–D)** Margalef, Shannon-Wiener, Simpson, Pielou indices in tree layer. **(E–H)** Margalef, Shannon-Wiener, Simpson, Pielou indices in shrub layer. **(I–L)** Margalef, Shannon-Wiener, Simpson, Pielou indices in herb layer.

Quadratic regression analyses further revealed layer-specific responses to elevation, with the quadratic models consistently showing higher R^2^ values than the linear models across all variable combinations (**Figure 3**). In the shrub layer, all *α*-diversity indces showed significant unimodal relationships with elevation (P < 0.05), with diversity increasing from low elevation to approximately 1000 m and decreasing thereafter. In the herb layer, only the Margalef index exhibited a significant unimodal pattern (P < 0.05), whereas the Shannon–Wiener, Simpson, and Pielou indices showed no significant relationships (P > 0.05). In the tree layer, none of the diversity indices showed significant linear or unimodal relationships with elevation (P > 0.05), although the Margalef index exhibited a decreasing trend. These results indicate that *α*-diversity responses to elevation are strongly dependent on forest layer, with the shrub layer showing the most pronounced elevational pattern.

### 3.4 Elevational patterns of *β*-diversity and species turnover

*β*-diversity of medicinal plants exhibited pronounced variation along the elevational gradient, with clear differences among forest layers. Species richness declined consistently with increasing elevation. Species turnover, quantified using the Cody index (*β_C_*), showed strong elevational patterns and marked differences among forest layers (**Table 4**). The shrub layer exhibited the highest turnover rates, with *β_C_* values ranging up to 59.50, particularly between 450–750 m and 750–1000 m, indicating rapid species replacement at low to mid elevations. The herb layer showed intermediate turnover, with *β_C_* values exceeding 45 at elevations below 1250 m and decreasing to moderate levels (approximately 30–33) at higher elevations. In contrast, the tree layer displayed relatively low and stable turnover with *β_C_* range from 20.00 to 23.50, suggesting limited compositional change along the gradient. In addition, the number of common species between elevational gradients showed a declining trend with increasing elevational distance. The tree layer exhibited relatively low and stable numbers of common species, whereas the shrub and herb layers showed higher but more variable values, particularly at lower elevations. This pattern indicates that species composition becomes increasingly distinct with elevation.

**Table 4:** Importance values of dominant species in different forest layers at different elevational gradients.

Forest layer	Elevational gradient/m	450–750	750–1000	1000–1250	1250–1500	1500–1800
Tree	450–750	0.00	23.50	30.50	33.00	26.00
	750–1000	22	0.00	21.00	26.50	18.50
	1000–1250	17	20	0.00	22.50	21.50
	1250–1500	14	14	20	0.00	20.00
	1500–1800	9	10	9	10	0.00
Shrub	450–750	0.00	59.50	77.00	79.50	78.00
	750–1000	66	0.00	52.50	61.00	63.50
	1000–1250	56	68	0.00	51.50	63.00
	1250–1500	40	46	63	0.00	49.50
	1500–1800	20	22	30	30	0.00
Herb	450–750	0.00	46.50	45.00	49.50	50.50
	750–1000	30	0.00	48.50	46.00	50.00
	1000–1250	24	21	0.00	33.50	32.50
	1250–1500	14	18	23	0.00	33.00
	1500–1800	17	18	28	22	0.00

The upper right corner of the table shows the Cody index value, and the lower right corner shows the number of common species.

**Figure 4:**
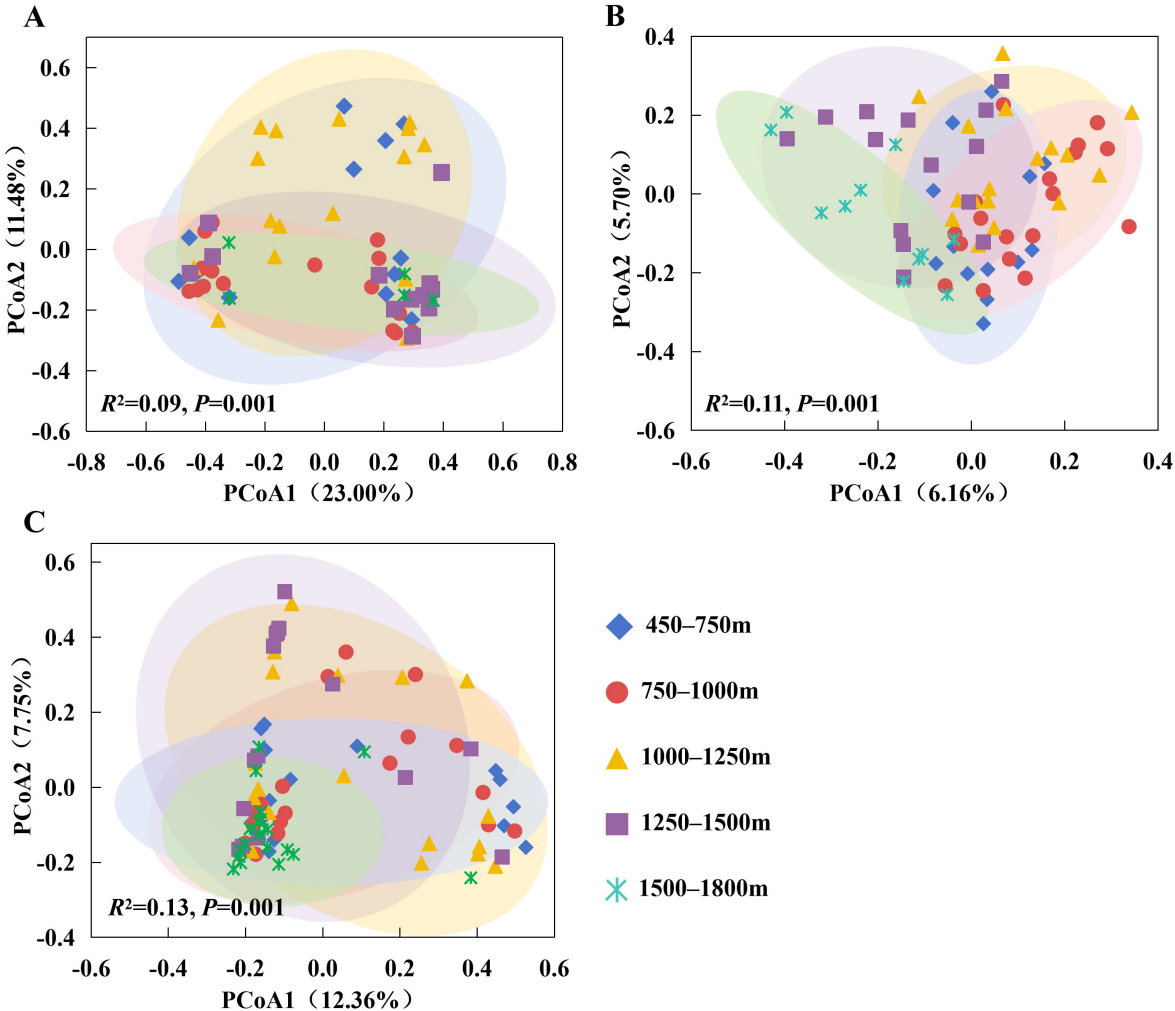
*β*-diversity of **(A)** tree layer, **(B)** shrub layer and **(C)** herb layer at different elevations based on principal co-ordinates analysis (PCoA)

Principal coordinate analysis (PCoA) further revealed significant effects of elevation on community composition (**Figure 4**). However, the strength of this effect differed among layers. The herb layer showed the highest sensitivity to elevation (R² = 0.13), followed by the shrub layer (R² = 0.11), whereas the tree layer exhibited the weakest response (R² = 0.09). Clear separation among elevational zones was observed in the shrub and herb layers. In the shrub layer, communities at 750–1000 m and 1250–1500 m were distinctly separated, indicating strong compositional differentiation. The 1000–1250 m zone occupied a broad ordination space and overlapped with multiple adjacent zones, suggesting its role as a transitional zone. In the herb layer, communities at 750–1250 m showed substantial overlap, reflecting continuous species turnover across mid-elevations, whereas the 1500–1800 m communities were more clustered, indicating reduced compositional heterogeneity at high elevations. In contrast, the tree layer showed considerable overlap among communities across elevational zones. The 1500–1800 m communities were largely encompassed within the distribution of 1250–1500 m and 750–1000 m communities, and substantial overlap was observed between 450–750 m and 1000–1250 m. These patterns indicate weak elevational structuring of tree-layer composition. Overall, β-diversity patterns indicate that community differentiation along the elevational gradient is strongest in the shrub and herb layers, while the tree layer exhibits relatively stable composition. The mid-elevation range (1000–1250 m) represents a key transition zone in community turnover.

### 3.5 Relationships between diversity and environmental factors

**Figure 5:**
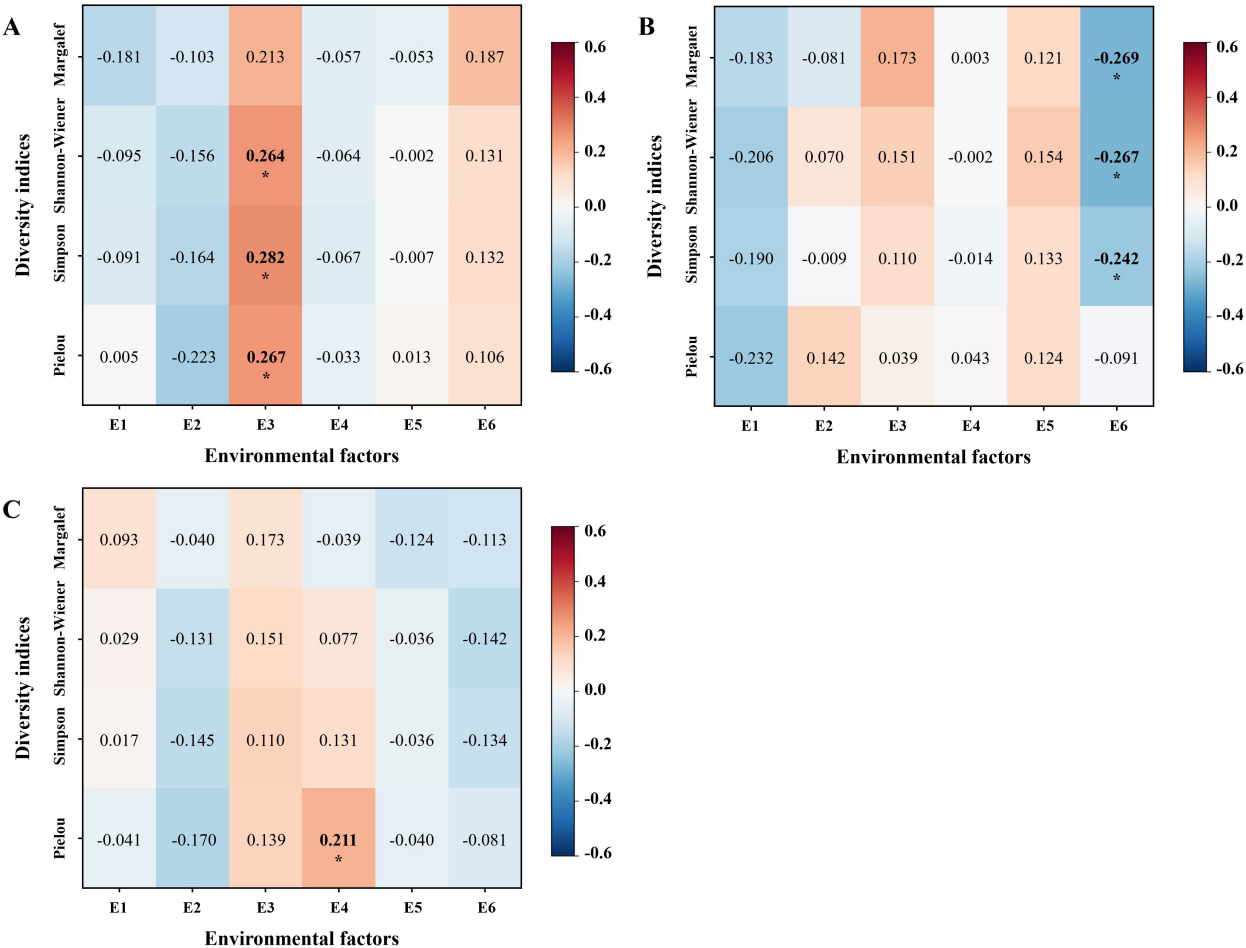
Heatmap of the Spearman rank correlation between *α*-diversity and environmental factors in **(A)** tree layer, **(B)** shrub layer and **(C)** herb layer. E1: Elevational gradient/m; E2: Latitude/°; E3: Longitude/°; E4: Slope/°; E5: Aspect; E6: Canopy closure/%. *P<0.05.

Spearman rank correlation analysis showed that some environmental factors were significantly associated with *α*-diversity across forest layers (**Figure 5**). In the tree layer, longitude was the only significantly influential factor, presenting significant positive correlations with the Shannon–Wiener, Simpson, and Pielou indices (P < 0.05), with correlation coefficients of 0.264, 0.282, and 0.267, respectively. In the shrub layer, canopy closure was significantly negatively correlated with the Shannon–Wiener, Simpson, and Pielou indices (P < 0.05), with correlation coefficients of –0.269, –0.267, and –0.242, respectively. Elevation was marginally correlated with the Pielou index (P = 0.051) with a slight negative tendency. In the herb layer, aspect was significantly positively correlated only with the Pielou index (P < 0.05), and canopy closure showed a marginally negative correlation with the Pielou index (P = 0.081). Overall, elevation, longitude and aspect had no significant correlations with *α*-diversity across all forest layers (P > 0.05).

**Figure 6:**
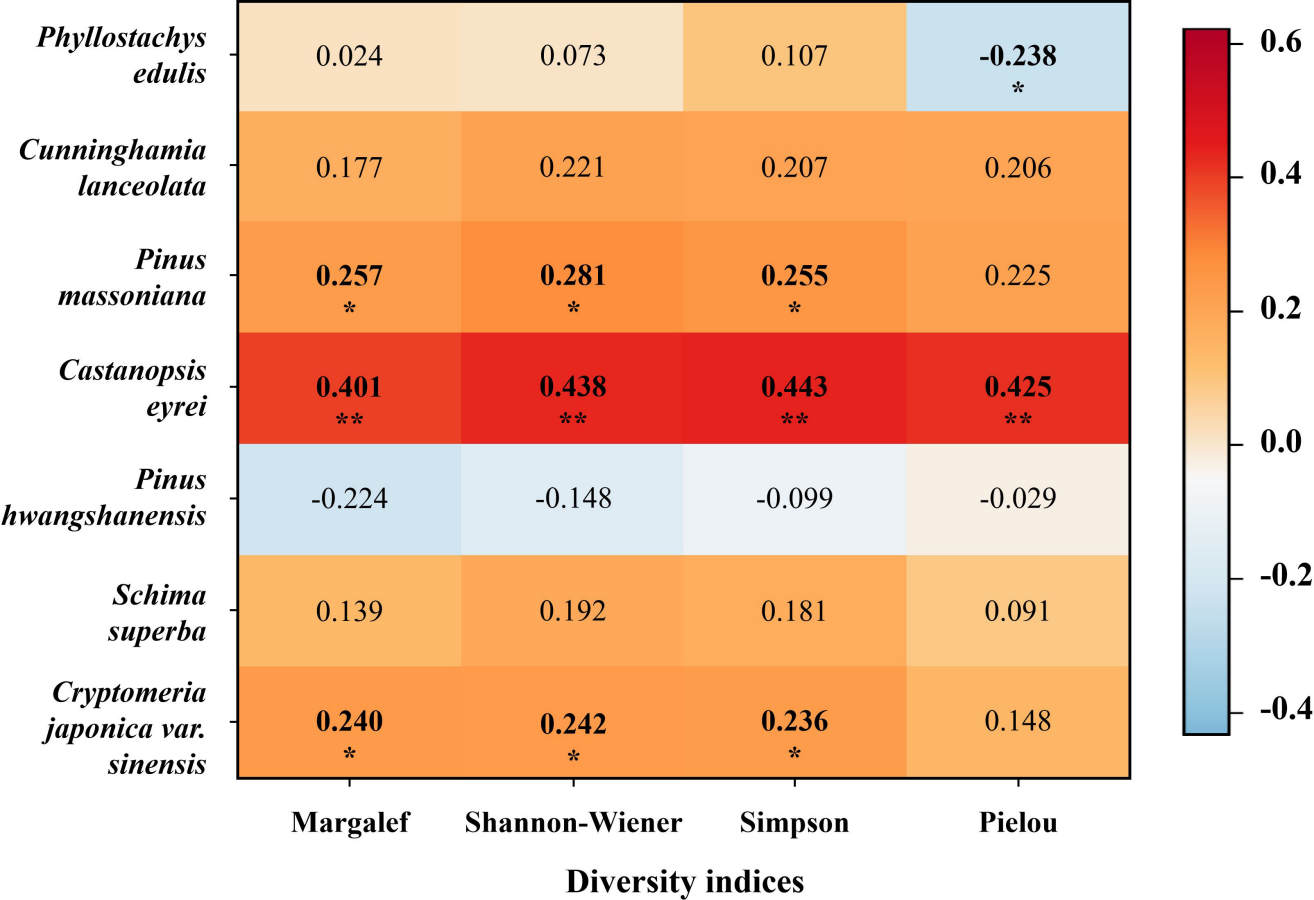
Heatmap of the Spearman rank correlation between α-diversity indices and environmental factors in shrub layers. *P<0.05, **P<0.01.

The relationships between dominant tree species and understory diversity revealed pronounced species-specific effects in the shrub layer (**Figure 6**). *Phyllostachys edulis* was the only dominant species showing a significant negative correlation with the Pielou index (correlation coefficient = –0.238). In contrast, *Pinus massoniana* and *Cryptomeria japonica* var. *sinensis* showed significant positive correlations with the Margalef, Shannon–Wiener, and Simpson indices (P < 0.05). The correlation coefficients were consistently higher for *Pinus massoniana*. Moreover, *Castanopsis eyrei* showed highly significant positive correlations with all diversity indices (P < 0.01), with correlation coefficients of 0.401, 0.438, 0.443, and 0.425, respectively. Overall, these results indicate that canopy structure exerts a stronger influence on medicinal plant diversity than topographic factors, and that the effects of dominant tree species on shrub-layer diversity are layer-dependent.

## 4 Discussion

### 4.1 Vertical stratification and elevational patterns of medicinal plant diversity

Our study demonstrates that medical plant diversity in subtropical montane ecosystems is jointly shaped by elevational gradients and vertical forest stratification, with clear layer-dependent responses. Species richness and *α*-diversity consistently followed the order shrub layer > herb layer > tree layer, indicating that understory layers contribute more to overall medicinal plant diversity. This pattern can be attributed to differences in life-history strategies and resource-use efficiency among layers (Díaz et al., 2016; Murphy et al., 2022). Shrub and herb species typically exhibit shorter life cycles, higher reproductive rates, and greater sensitivity to microenvironmental variation, enabling them to exploit heterogeneous light and soil conditions in the understory (Barbier et al., 2008). In contrast, tree-layer diversity is constrained by stronger competition for light and space, as well as longer life spans, which buffer short-term environmental fluctuations (Salguero-Gómez et al., 2015; Yang et al., 2025). These findings are consistent with previous studies showing that understory vegetation plays a critical role in maintaining forest biodiversity and ecosystem functioning (Huo et al., 2014). Moreover, recent evidence suggests that shrub-layer diversity contributes substantially to forest productivity, with effects comparable to those of the tree layer, highlighting the ecological importance of understory vegetation in maintaining biodiversity and ecosystem processes (Chen et al., 2025a) Importantly, our results highlight that vertical stratification is not merely a structural feature but a key ecological dimension regulating diversity patterns. Ignoring layer-specific responses may obscure important mechanisms underlying biodiversity distribution along environmental gradients (Wang et al., 2021).

### 4.2 Mechanisms underlying *α*-diversity variation along elevation

The observed elevational patterns of *α*-diversity varied among forest layers in Meihua mountain, reflecting the interaction of multiple ecological processes. In the shrub layer, all diversity indices exhibited significant unimodal relationships with elevation, peaking at mid-elevations (~1000 m). This pattern is consistent with the mid-domain effect, where geometric constraints and overlapping species ranges lead to maximum richness at intermediate elevations (Colwell and Lees, 2000). At the same time, favorable hydrothermal conditions and habitat heterogeneity at mid-elevations likely enhance species coexistence, reinforcing this peak (Thakur et al., 2022). The herb layer showed a weaker unimodal pattern in Meihua mountain, with only species richness (Margalef index) displaying significant variation. This suggests that herb-layer diversity is more strongly influenced by fine-scale environmental heterogeneity, such as light availability and soil moisture, rather than broad-scale elevational gradients (Sabatini et al., 2014a; Radhamoni et al., 2023). The relatively continuous species turnover observed in mid-elevation zones further supports this interpretation. In contrast, the tree layer exhibited no significant elevational trend in *α*-diversity. This stability likely reflects the dominance of long-lived species with broad ecological tolerances, which are less responsive to short-term environmental variation (Guo et al., 2013). Such patterns indicate that environmental filtering operates more strongly on understory layers, while tree-layer diversity is shaped by longer-term successional and historical processes (Candeias and Fraterrigo, 2020; Chen et al., 2025b). These layer-specific patterns suggest that the effects of elevation on *α*-diversity are not uniform but are mediated by both vertical stratification and environmental constraints operating at different spatial scales (Sabatini et al., 2014b). At low elevations, stronger human disturbance and the prevalence of plantation forests (e.g., *Cunninghamia lanceolata* and *Phyllostachys edulis*) may further influence diversity patterns by altering habitat conditions and competitive dynamics (Sun et al., 2024; Wang et al., 2025). At high elevations, harsher climatic conditions act as environmental filters, limiting species establishment and reducing diversity.

### 4.3 Drivers of *β*-diversity and community turnover across forest layers

*β*-diversity analysis revealed pronounced differences among forest layers, with the shrub layer exhibiting the highest species turnover, followed by the herb layer, and the tree layer showing the lowest turnover. These results indicate that community composition in understory layers is more responsive to elevational gradients, whereas tree-layer composition remains relatively stable (Deng et al., 2025). High turnover in the shrub layer suggests rapid species replacement along environmental gradients, likely driven by strong sensitivity to changes in temperature, moisture, and light availability. In contrast, the lower turnover in the tree layer reflects the persistence of dominant species and slower community dynamics (Fang et al., 2024). The identification of a mid-elevation transition zone (1000–1250 m) further highlights the importance of environmental gradients in structuring community composition. This zone showed the greatest overlap in species composition and the widest ordination space in PCoA, indicating active species exchange and coexistence. Similar transition zones have been reported in other mountain ecosystems and are often associated with high *β*-diversity due to overlapping ecological niches and gradual environmental shifts (Ivanova and Soukhovolsky, 2016; Jiang et al., 2023). Compared with studies in temperate and alpine regions, where herb-layer turnover is often highest (Yang et al., 2016), our findings emphasize that the relative importance of vertical stratification in forests in driving *β*-diversity is layer-specific, likely reflecting differences in climate, vegetation structure, and disturbance regimes (Chai et al., 2023).

### 4.4 Combined effects of canopy structure and environmental factors on *α*-diversity

Among the environmental variables examined, canopy closure remained an important factor influencing α-diversity, with negative effects observed particularly in the shrub layer (Vallé et al., 2025). This finding underscores the central role of forest structural attributes in regulating understory biodiversity (De Benedictis et al., 2026). Dense canopy cover reduces light availability and limits the establishment and growth of understory species, leading to lower diversity. Conversely, more open canopies enhance light heterogeneity and resource availability, promoting species coexistence (Majasalmi and Rautiainen, 2020). However, the effects of canopy closure were not consistent across all forest layers. No significant relationship was detected in the herb layer, suggesting that herbaceous species are not uniformly constrained by canopy-mediated light limitation (Han et al., 2023). Instead, herb-layer diversity was positively associated with slope, with higher diversity observed on steeper terrain. This pattern may be related to reduced competitive exclusion, increased microhabitat heterogeneity, and improved drainage conditions on steeper slopes (Tian et al., 2023). In addition, tree-layer diversity was positively correlated with latitude, indicating a spatial gradient in species richness, with higher diversity toward the eastern part of the study area. This pattern may reflect broader environmental gradients, such as regional climatic variation or biogeographic transitions, which influence canopy composition and diversity (Kreft and Jetz, 2007). The effects of dominant tree species further support the importance of canopy-related characteristics. *Phyllostachys edulis* was negatively associated with shrub-layer diversity, likely due to dense canopies and strong competitive ability (Song et al., 2017). In contrast, *Pinus massoniana* (Liu et al., 2021) and *Cryptomeria japonica* var. *sinensis* (Kamei et al., 2025) showed positive associations, possibly reflecting more open canopy structures that facilitate understory growth (Dormann et al., 2020). Overall, these results suggest that canopy structure remains a key factor influencing understory diversity, particularly in the shrub layer, while topographic and spatial factors exert additional, layer-specific effects across the forest ecosystem.

It should be noted that historical human activities, such as the collection of medicinal plants, may have influenced the current distribution and abundance of certain species. Although the study area is now under strict protection, legacy effects of past disturbances cannot be entirely excluded. Future studies incorporating land-use history or ethnobotanical information would help to better disentangle natural and anthropogenic drivers of diversity patterns.

## 5 Conclusion

This study demonstrates that medicinal plant diversity in Meihua Mountain is jointly shaped by elevational gradients and vertical forest stratification, with clear layer-dependent patterns. Species richness and *α*-diversity consistently followed the order shrub > herb > tree layer, indicating that understory vegetation contributes disproportionately to overall diversity. Diversity responses to elevation differed markedly among layers. The shrub layer exhibited a significant unimodal pattern, while the herb layer showed a weaker mid-elevation peak, and the tree layer displayed no clear trend. These contrasting patterns reflect differences in life-history strategies and the varying strength of environmental filtering across forest layers. *β*-diversity analysis further revealed that species turnover was highest in the shrub layer and lowest in the tree layer, with a distinct transition zone at mid-elevations. This indicates greater sensitivity of understory communities to environmental gradients, whereas tree-layer composition remains relatively stable. Among environmental factors, canopy closure showed a significant negative association with shrub-layer *α*-diversity. In addition, longitude and slope exerted significant positive effects on tree- and herb-layer diversity, respectively. These findings suggest that biodiversity patterns are shaped by a combination of canopy structure, topographic conditions, and spatial gradients, with their relative importance varying across forest layers. Overall, these findings highlight vertical stratification as a key dimension of biodiversity organization and provide new insights into diversity maintenance mechanisms in subtropical montane ecosystems. Nevertheless, this study is based on a single mountain system and primarily considers structural and topographic variables, without explicitly accounting for potential legacy effects of past human activities. Future research incorporating broader geographic scales and additional environmental factors (e.g., soil properties and microclimate) will further improve our understanding of the mechanisms driving medicinal plant diversity.

## Data Availability

The original contributions presented in the study are included in the article/**Supplementary Material**. Further inquiries can be directed to the corresponding authors.
