# Supplementary material for: Diversity patterns of medicinal plants along elevational gradients across forest layers in Meihua Mountains, Fujian
Source: Front Plant Sci. 2026 May 29;17:1856248. doi: 10.3389/fpls.2026.1856248 (PMC13260540; doi:10.3389/fpls.2026.1856248)
Supplement: Supplementary file 1 [file Table1.docx]

Supplementary Material

**Supplementary Table 1 The Shapiro-Wilk test of α-diversity indices across forest layers**

| Forest Layer | Elevational gradient/m | Index | W statistic | P value | Normality |
| --- | --- | --- | --- | --- | --- |
| Tree | 450–750 | Margalef | 0.965 | 0.892 | Yes |
|  |  | Shannon–Wiener | 0.943 | 0.668 | Yes |
|  |  | Simpson | 0.929 | 0.510 | Yes |
|  |  | Pielou | 0.936 | 0.590 | Yes |
|  | 750–1000 | Margalef | 0.957 | 0.585 | Yes |
|  |  | Shannon–Wiener | 0.932 | 0.282 | Yes |
|  |  | Simpson | 0.911 | 0.136 | Yes |
|  |  | Pielou | 0.904 | 0.103 | Yes |
|  | 1000–1250 | Margalef | 0.901 | 0.199 | Yes |
|  |  | Shannon–Wiener | 0.887 | 0.134 | Yes |
|  |  | Simpson | 0.864 | 0.076 | Yes |
|  |  | Pielou | 0.848 | 0.051 | Yes |
|  | 1250–1500 | Margalef | 0.923 | 0.457 | Yes |
|  |  | Shannon–Wiener | 0.898 | 0.294 | Yes |
|  |  | Simpson | 0.875 | 0.189 | Yes |
|  |  | Pielou | 0.857 | 0.125 | Yes |
|  | 1500–1800 | Margalef | 0.902 | 0.488 | Yes |
|  |  | Shannon–Wiener | 0.883 | 0.366 | Yes |
|  |  | Simpson | 0.865 | 0.278 | Yes |
|  |  | Pielou | 0.844 | 0.202 | Yes |
| Shrub | 450–750 | Margalef | 0.923 | 0.309 | Yes |
|  |  | Shannon–Wiener | 0.972 | 0.926 | Yes |
|  |  | Simpson | 0.877 | 0.081 | Yes |
|  |  | Pielou | 0.862 | 0.052 | Yes |
|  | 750–1000 | Margalef | 0.981 | 0.962 | Yes |
|  |  | Shannon–Wiener | 0.926 | 0.168 | Yes |
|  |  | Simpson | 0.693 | 0.000 | No |
|  |  | Pielou | 0.673 | 0.000 | No |
|  | 1000–1250 | Margalef | 0.951 | 0.467 | Yes |
|  |  | Shannon–Wiener | 0.831 | 0.006 | No |
|  |  | Simpson | 0.653 | 0.000 | No |
|  |  | Pielou | 0.705 | 0.000 | No |
|  | 1250–1500 | Margalef | 0.892 | 0.104 | Yes |
|  |  | Shannon–Wiener | 0.948 | 0.570 | Yes |
|  |  | Simpson | 0.842 | 0.022 | No |
|  |  | Pielou | 0.784 | 0.005 | No |
|  | 1500–1800 | Margalef | 0.949 | 0.619 | Yes |
|  |  | Shannon–Wiener | 0.946 | 0.573 | Yes |
|  |  | Simpson | 0.904 | 0.179 | Yes |
|  |  | Pielou | 0.912 | 0.226 | Yes |
| Herb | 450–750 | Margalef | 0.978 | 0.976 | Yes |
|  |  | Shannon–Wiener | 0.906 | 0.191 | Yes |
|  |  | Simpson | 0.887 | 0.107 | Yes |
|  |  | Pielou | 0.865 | 0.056 | Yes |
|  | 750–1000 | Margalef | 0.950 | 0.417 | Yes |
|  |  | Shannon–Wiener | 0.894 | 0.045 | No |
|  |  | Simpson | 0.736 | 0.000 | No |
|  |  | Pielou | 0.713 | 0.000 | No |
|  | 1000–1250 | Margalef | 0.894 | 0.054 | Yes |
|  |  | Shannon–Wiener | 0.907 | 0.090 | Yes |
|  |  | Simpson | 0.852 | 0.011 | No |
|  |  | Pielou | 0.856 | 0.013 | No |
|  | 1250–1500 | Margalef | 0.919 | 0.244 | Yes |
|  |  | Shannon–Wiener | 0.924 | 0.285 | Yes |
|  |  | Simpson | 0.884 | 0.080 | Yes |
|  |  | Pielou | 0.770 | 0.003 | No |
|  | 1500–1800 | Margalef | 0.939 | 0.479 | Yes |
|  |  | Shannon–Wiener | 0.912 | 0.227 | Yes |
|  |  | Simpson | 0.877 | 0.080 | Yes |
|  |  | Pielou | 0.931 | 0.388 | Yes |
